# Beyond Autonomy, Competence, and Relatedness: A Comprehensive Scale for Basic Psychological Needs and Novelty in Exercise

**DOI:** 10.3390/healthcare14080995

**Published:** 2026-04-10

**Authors:** Vera Bártolo, Miguel Jacinto, Nuno Amaro, Raúl Antunes, Rui Matos, Nuno Couto, Luís Cid, Pedro Duarte-Mendes, Filipe Rodrigues, Diogo Monteiro

**Affiliations:** 1 ESECS, Polytechnic University of Leiria, 2411-901 Leiria, Portugal; veraquintela@gmail.com (V.B.); miguel.s.jacinto@ipleiria.pt (M.J.); nuno.amaro@ipleiria.pt (N.A.); raul.antunes@ipleiria.pt (R.A.); rui.matos@ipleiria.pt (R.M.); filipe.rodrigues@ipleiria.pt (F.R.); 2 Research Centre in Sport, Health and Human Development (CIDESD), 2411-901 Leiria, Portugal; ncouto@esdrm.ipsantarem.pt (N.C.);; 3 Sport Sciences School of Rio Maior (ESDRM), Santarém Polytechnic University, 2040-413 Santarém, Portugal; 4 Department of Sports and Well-Being, Polytechnic Institute of Castelo Branco, 6000-084 Castelo Branco, Portugal; 5 Sport Physical Activity and Health Research & Innovation Center (SPRINT), 6000-084 Castelo Branco, Portugal

**Keywords:** motivation, self-determination theory, novelty, enjoyment, satisfaction with life, exercise

## Abstract

**Background/Objectives:** Grounded in Self-Determination Theory, this study aimed to translate and validate the Basic Psychological Need Satisfaction and Frustration Scale (BPNSFS), including the Novelty dimension, within the Portuguese exercise context. Given the emerging evidence of novelty as a potential candidate for a basic psychological need, this research examined the psychometric properties and temporal stability. Furthermore, this study explored the nomological validity of these constructs regarding exercise enjoyment and satisfaction with life. **Methods:** The sample comprised 500 gym-goers (263 females; 237 males), aged between 18 and 65 years (M = 33.76; SD = 12.94). Confirmatory Factor Analysis and Exploratory Structural Equation Modeling were employed to compare the factor structure. Temporal stability was assessed through a test–retest procedure with a four-week interval (n = 50). **Results:** Exploratory Structural Equation Modeling provided a superior fit to the data (CFI = 0.98; TLI = 0.97; RMSEA = 0.04) compared to Confirmatory factor Analysis, supporting the distinctiveness of the eight dimensions. The instrument demonstrated strong internal consistency (composite reliability ranging from 0.78 to 0.90) and adequate discriminant validity. Path analysis revealed that novelty satisfaction was significantly associated with enjoyment and satisfaction with life. In reverse, novelty frustration was negatively associated with these well-being indicators. Intraclass correlation coefficients (0.75 to 0.83) confirmed robust temporal stability. **Conclusions:** These findings provide evidence that the Portuguese version of the BPNSFS, including the novelty dimension, is a psychometrically comprehensive instrument for the exercise context. The results support the inclusion of novelty as a relevant psychological need within Self-Determination Theory.

## 1. Introduction

The health benefits of physical activity in modifying chronic diseases and reducing premature mortality are extensively documented [1,2]. However, contemporary societies face a sedentary lifestyle: in the European Union, 45% of citizens report never exercising, a tendency that is even more pronounced in Portugal, where inactivity reaches 75% of the population [3]. In this public health context, understanding the motivational mechanisms that sustain regular exercise is an essential imperative for promoting healthy and productive lifestyles [1,2].

Motivation, as a primary regulator of human action [4,5], is robustly framed within Self-Determination Theory [6]. Moving beyond a unitary view of motivation, Self-Determination Theory proposes an organismic perspective that differentiates the quality of motivation along a self-determination continuum: ranging from amotivation (the absence of intent) and extrinsic regulation (driven by external rewards or pressures) to intrinsic motivation, characterized by inherent interest and enjoyment in the activity [7]. In the exercise domain, the process of internalization, the transition from controlled to autonomous regulation, is a critical determinant for long-term adherence [8,9], with motivational quality serving as a superior predictor of behavioral change compared to mere motivational intensity [10].

At the core of Self-Determination Theory, Basic Psychological Needs Theory posits that human growth and eudaimonic well-being depend on the satisfaction of three innate and universal needs: autonomy (a sense of volition and internal endorsement), competence (perceived effectiveness in mastering tasks), and relatedness (a sense of connection and belonging with others) [9,11,12]. While the satisfaction of these needs catalyzes autonomous motivation and psychological integrity, their frustration, a concept distinct from low satisfaction, is associated with diminished functioning and psychological well-being [9]. Although Self-Determination Theory includes six micro-theories that explain various sides of human behavior [9], the interplay between need satisfaction and frustration remains the most robust predictive link for motivational outcomes in exercise settings.

### 1.1. Novelty

Self-Determination Theory has been widely studied, and recently, a proposal for a fourth basic psychological need, novelty, has emerged. Several authors investigating motivation and human behavior have included variables such as curiosity, perceived variety, or sensation seeking in their research, consistently relating the search for novelty to the avoidance of boredom and the achievement of psychological well-being [13,14,15].

However, for a construct to be considered a basic psychological need, it must fulfill specific criteria: it must be independent, essential, universal, and demonstrate an association with functioning that extends beyond immediate psychological responses, with negative consequences occurring when it is frustrated [16,17].

In this context, González-Cutre et al. [18] proposed novelty as a new basic psychological need, defining it as the “need to experience something not previously experienced or that deviates from everyday routine”. These authors developed a scale to measure novelty satisfaction by adding six new items to the existing Basic Psychological Need Satisfaction and Frustration Scale. Their findings indicated that the correlations between novelty and the other three needs were consistent with the inter-correlations typically observed among autonomy, competence, and relatedness [18].

Expanding this line of research, Trigueros et al. [19] introduced a scale to measure novelty frustration, with results reinforcing novelty as a solid candidate for a fourth need. Simultaneously, González-Cutre et al. [20] continued to investigate the importance of novelty for multiple positive outcomes, finding promising results [8,9,10]. The novelty construct has also shown invariance across genders, aligning with studies on the original psychological needs and supporting its universality [6,19,21,22,23,24].

Novelty satisfaction has been linked to several positive outcomes such as a positive association with autonomous motivation, enjoyment, well-being, and life satisfaction [25,26,27]. While several studies provide evidence that novelty meets the criteria of a basic psychological need, finding a good fit for the novelty scale in different countries and contexts, particularly in physical education, many authors have noted the cross-sectional nature of these studies as a limitation. This methodological design excludes conclusions about causality and limits the ability to analyze whether novelty satisfaction is associated with positive consequences over extended periods [16,25,26,28,29,30].

### 1.2. Enjoyment, Satisfaction with Life and Basic Psychological Needs

Adherence to exercise remains a complex challenge, as many individuals initiate programs only to withdraw shortly thereafter. Consequently, it is essential to understand how basic psychological needs relate to enjoyment, a key emotional component that determines whether a behavior is maintained or abandoned [31,32]. Enjoyment is defined as a positive affective response to physical activity characterized by feelings of pleasure and interest [33]. Within the Self-Determination Theory framework, high levels of intrinsic motivation are intrinsically linked to enjoyment, which acts as both a consequence of and a motivator for self-determined engagement [11,34]. Because enjoyment strongly predicts long-term adherence [35], exploring its association with the satisfaction of psychological needs provides critical evidence for the construct validity of the novelty dimension [36].

Beyond immediate emotional responses, Self-Determination Theory explores the connection between need satisfaction and broader well-being [6]. Psychological research distinguishes between the hedonic perspective, which equates well-being with the pursuit of pleasure and pain avoidance [6,37], and the eudaimonic perspective, which views well-being as the realization of human potential and the ‘true self’ [6,11,37]. Self-Determination Theory aligns with the eudaimonic approach, asserting that being fully functioning, through the satisfaction of autonomy, competence, and relatedness, is the primary driver of both subjective and eudaimonic well-being [6,37].

Emerging evidence suggests that novelty plays a similar role in this process. Studies have demonstrated that novelty satisfaction positively predicts autonomous motivation, exercise habits, and life satisfaction [18,20,25,26,27]. Specifically, work by González-Cutre [18,20] has shown that novelty is associated with psychological integrity, vitality, and flow, meeting the essential criteria to be considered a candidate basic psychological need [11,18,20]. Therefore, investigating the relationship between novelty and outcomes such as enjoyment and life satisfaction is crucial to confirm if its predictive power aligns with the established literature on the three traditional needs.

### 1.3. Current Study

The construct of novelty has emerged as a significant candidate for a basic psychological need within Self-Determination Theory. Novelty satisfaction was first validated by González-Cutre et al. [18] using a 6-item scale that demonstrated robust psychometric properties [CFI = 0.99; IFI = 0.99; RMSEA = 0.07 (90% CI = 0.04–0.10); SRMR = 0.02]. Subsequent research by the same authors expanded this to an eight-factor model, integrating both novelty satisfaction and frustration subscales, which also yielded an acceptable fit [CFI = 0.94; TLI = 0.93; RMSEA = 0.05 (90% CI 0.04–0.05); SRMR = 0.04]. Furthermore, the 5-item novelty frustration subscale was tested with factor loadings exceeding 0.68 [18,20]. Empirical evidence has shown that novelty satisfaction is positively associated with life satisfaction, vitality, enjoyment, and flow [18,20]. In contrast, research by Birdsell [27] demonstrated that the frustration of novelty, treated as a basic need, is negatively associated with satisfaction and engagement, while being linked to reduced well-being. These findings highlight the conceptual robustness of novelty across domains like sport and education.

The present study aims to reinforce this construct within the Portuguese exercise context by adapting and validating the Basic Psychological Need Satisfaction and Frustration Scale integrating Novelty (BPNSFNS). By consolidating the novelty construct, this research provides a validated instrument to better understand exercise motivation and promote lasting behavioral change. To achieve a comprehensive psychometric evaluation, the specific objectives of this study are: (a) to verify the temporal stability of the scale through a four-week test–retest procedure; (b) to evaluate the factor structure of the BPNSFNS by comparing the traditional confirmatory factor analysis with the exploratory structural equation modeling approach to account for conceptual overlaps; (c) to examine the internal consistency and convergent validity of the eight dimensions using composite reliability coefficients and average variance extracted; (d) to assess discriminant validity through the Heterotrait–Monotrait ratio of correlations, ensuring the distinctiveness of novelty in relation to autonomy, competence, and relatedness; (e) to test the nomological validity of the instrument by analyzing the positive associations of need satisfaction and frustration on exercise enjoyment and life satisfaction.

## 2. Materials and Methods

### 2.1. Participants

The sample size for the structural equation modeling and confirmatory factor analyses was determined using the a priori sample size calculator for structural equation models [38]. The objective was to compute the minimum sample size required given the number of observed and latent variables in the model, the anticipated effect size, and the desired probability and statistical power levels. Considering an anticipated effect size of 0.25, a desired statistical power level of 0.95, a probability level of 0.05, 8 latent variables (i.e., the eight basic psychological needs), and 35 observed variables, the calculator returned a minimum sample size of 89 for the model structure and a minimum sample size of 391 to detect the specified effect. Thus, the recommended minimum sample size was 391. Furthermore, to ensure adequate sample size, we also followed the recommendations for multivariate data analysis [39,40]. Specifically, Hair et al. [39] and Kline [40] recommend a minimum ratio of 10 participants per observed variable (10:1) for confirmatory factor analysis and structural equation modelling. Given that our measurement model comprises 35 observed variables from the BPNSFNS scale, a minimum of 350 participants would be required.

To be included in the present study, individuals had to meet the following inclusion criteria: (i) be aged between 18 and 65 years, aiming to capture a wide variance and representativeness of the adult lifespan; (ii) be active gym members with a current and valid membership; (iii) be fluent in Portuguese to ensure a clear and accurate comprehension of the questionnaire items; and (iv) provide written informed consent, agreeing to participate voluntarily.

### 2.2. Procedures

Approval of the research was obtained from the Ethical Committee (reference number: 17/2024) of the first author’s institution. Following ethical approval, the researchers proceeded with the translation and adaptation of the instrument. It is important to note that the original Basic Psychological Need Satisfaction and Frustration Scale had already been previously validated for the Portuguese context. Therefore, this methodological step focused specifically on the translation of the need for novelty items from English to Portuguese and their subsequent integration into the new scale to form the BPNSFNS. This adaptation followed the committee approach methodology [41], as recommended by Banville et al. [42] and Cid et al. [43]. The process involved five steps: (i) Preliminary Translation: Researchers, with the assistance of three translators holding advanced qualifications in the English and Portuguese languages, created the first version of the novelty items; (ii) First Evaluation Panel: Four specialists from diverse fields, namely English Portuguese Languages, Psychology, Sports Psychology, and Sports Sciences, individually analyzed the initial version. Minor modifications were made based on their suggestions; (iii) Second Evaluation Panel: The revised version was reviewed by a new panel of four specialists in Psychology, Sports Psychology, and Sports Sciences. This group collaboratively evaluated all items and reached consensus on the third version of the questionnaire; (iv) Pilot Study: A group of 50 exercisers completed the revised version to ensure the clarity and comprehensibility of the items; (v) Final Revision: Two Portuguese language teachers reviewed the final version to address syntax, spelling, and grammatical errors. This thorough process ensured the translation’s quality and cultural appropriateness.

Subsequently, the researchers contacted the managers of various fitness centers to explain the study’s overall objectives and request formal permission to collect data from their clients. With consent granted, the authors were present at these diverse fitness facilities during different times of the day to invite individuals to participate. Gym members were approached individually in the reception. Participation was entirely voluntary, and data collection took place in person using a paper-and-pencil questionnaire format before participants began their exercise sessions. To ensure privacy and reduce bias, participants were provided with a quiet space to complete the survey independently. No monetary compensation was provided for their involvement.

### 2.3. Instruments

Basic Psychological Need Satisfaction and Frustration Scale integrating Novelty. The Portuguese version of the Basic Psychological Need Satisfaction and Frustration Scale [24] was used to measure the satisfaction and frustration of the three traditional basic psychological needs: autonomy, competence, and relatedness. This 24-item base questionnaire is rated on a 5-point Likert scale ranging from 1 (“totally disagree”) to 5 (“totally agree”). It assesses six dimensions, including autonomy satisfaction (e.g., “I feel a sense of choice and freedom in the things I undertake”), competence satisfaction (e.g., “I feel confident that I can do things well”), relatedness satisfaction (e.g., “I feel connected with people who care for me, and for whom I care”), autonomy frustration (e.g., “I feel forced to do many things I wouldn’t choose to do”), competence frustration (e.g., “I feel disappointed with my performance”), and relatedness frustration (e.g., “I feel excluded from the group I want to belong to”). To construct the comprehensive version (BPNSFNS) utilized in this study, the dimensions of novelty satisfaction and novelty frustration [18,20] were translated and added to the original instrument. This integration comprised 11 new items, six for satisfaction (e.g., “I feel I do novel things”) and five for frustration (e.g., “I feel I am constantly doing the same routine things”), following the same 5-point response format, which resulted in a final 35-item scale assessing eight dimensions.

Enjoyment. The Portuguese version of the Physical Activity Enjoyment Scale [44] was used to measure participants’ enjoyment during exercise. This 8-item scale evaluates the level of agreement with statements about exercise enjoyment (e.g., “It is very stimulating”) using a 7-point Likert scale, ranging from 1 (“totally disagree”) to 7 (“totally agree”).

Satisfaction with Life. The Portuguese version of the Satisfaction with Life Scale [45] was used to assess participants’ overall cognitive judgment of their Satisfaction with life. The scale consists of five items (e.g., “I am satisfied with my life”), with responses rated on a 5-point Likert scale ranging from 1 (“strongly disagree”) to 5 (“strongly agree”).

### 2.4. Statistical Analysis

Data were initially imported into and analyzed using IBM SPSS Statistics for Windows, Version 31.0 (IBM Corp., Armonk, NY, USA). To assess the temporal reliability of the instrument (BPNSFNS), a test–retest procedure was conducted during the data collection phase using a convenience sample of 50 exercisers. Participants completed the questionnaire twice, with a time interval of four weeks between the first and second measurements. Temporal reliability was evaluated by calculating the Intraclass Correlation Coefficient for each factor, rather than for individual items. Analyzing factors is a recommended and useful approach for evaluating instruments with well-defined subscales, as the aggregate reliability of the items provides a more meaningful measure than item-level reliability [39]. The cutoff values applied to evaluate the ICC were as follows: <0.50 indicating poor reliability, 0.50 to 0.75 indicating moderate reliability, 0.75 to 0.90 indicating good reliability, and >0.90 indicating excellent reliability [46].

Data analysis was conducted using Mplus version 7.4 [47]. Given the ordinal nature of the 5-point Likert scale, the robust Weighted Least Squares Mean and Variance adjusted estimator were employed. First, since the original instrument has been previously validated, a confirmatory factor analysis was performed to evaluate the psychometric properties of the measurement model and verify if the new novelty satisfaction and frustration items adequately fit the established theoretical structure of the BPNSFNS. Furthermore, to examine the factor structure and address potential discriminant validity concerns related to conceptual overlaps between the basic psychological needs, an exploratory structural equation modeling approach was also evaluated. For all analyses, the guidelines provided by several authors [39,40,48,49] were followed. The following goodness-of-fit indexes were used: Comparative Fit Index (CFI), Tucker–Lewis Index (TLI), Root Mean Square Error of Approximation (RMSEA) with its 90% Confidence Interval (CI), and Weighted Root Mean Square Residual (WRMR). The cut-off values assumed were: CFI and TLI ≥ 0.90, with values ≥ 0.95 indicating excellent fit, and RMSEA ≤ 0.08 while a value ≤ 0.05 indicates a close or excellent fit. Given the ordinal nature of the data (5-point Likert scale) and the use of the Weighted Least Squares Mean and Variance adjusted estimator, the WRMR was evaluated instead of the Standardized Root Mean Square Residual (SRMR). For the WRMR, values ≤ 1.00 indicate a good model fit, as recommended for categorical data [14,30,37,38,39].

The psychometric evaluation of the BPNSFNS followed a systematic, multi-step procedure to ensure internal consistency and construct validity. Initially, the global fit of the measurement models was assessed using the previously defined goodness-of-fit indices. Following the confirmation of an adequate model fit, the individual parameters were analyzed. Internal consistency was evaluated using Raykov’s formula [50] to calculate composite reliability coefficients, with values ≥ 0.70 considered indicative of good reliability. To assess convergent validity, we examined both the factor loadings (λ) and the Average Variance Extracted (AVE). In line with Hair et al. [39], standardized factor loadings should ideally be ≥0.50, and the AVE should be ≥0.50 to ensure that the latent construct explains more than half of the variance of its indicators.

Discriminant validity was tested using two complementary approaches: (i) the Fornell and Larcker [51] criterion, which requires the AVE of each construct to be greater than the squared correlation (*r*^2^) with any other construct; and (ii) the Heterotrait–Monotrait ratio of correlations. According to Henseler et al. [52], Heterotrait–Monotrait values close to 1.0 indicate a lack of discriminant validity, with a recommended conservative threshold of ≤0.85 or a more liberal one of ≤0.90. In cases where discriminant validity issues emerged due to the high conceptual proximity of the basic psychological needs, we formally evaluated an exploratory structural equation modeling approach [53] to account for potential cross-loadings and provide a more accurate representation of the instrument’s structure.

The nomological validity of the BPNSFNS was examined through structural equation modeling. Nomological validity refers to the degree to which a construct behaves as expected within a system of related theoretical hypotheses. In this study, we assessed the relationships between the satisfaction and frustration of basic psychological needs, including novelty, with external outcomes: exercise enjoyment and satisfaction with life. The structural model was specified by entering all eight latent variables (four need satisfaction and four need frustration) simultaneously as independent variables. As recommended by Williams and MacKinnon [54], the significance of these standardized paths was evaluated using 95% Confidence Intervals (CI). Relationships were considered statistically significant when the 95% CI did not include zero, providing evidence that the constructs in the scale relate meaningfully to established well-being and motivational indicators in the exercise domain.

## 3. Results

The final sample consisted of 500 Portuguese fitness practitioners. Regarding gender distribution, 263 were female (52.6%) and 237 were male (47.4%). The participants’ ages ranged from 18 to 65 years (M = 33.76; SD = 12.94). In terms of exercise habits, most of the sample practiced bodybuilding/resistance training (49.6%), followed by group classes (38.8%), and personal training (11.6%). The average training weekly frequency was approximately 3.44 sessions (SD = 1.44) and an average session duration of 64.60 min (SD = 24.30). Detailed sociodemographic and exercise characteristics are presented in Table 1.

Results of the intraclass correlation coefficients (see Table 2) revealed good temporal reliability for most factors, with values ranging from 0.75 to 0.83. Specifically, Autonomy Satisfaction, Autonomy Frustration, Competence Frustration, Relatedness Satisfaction, Relatedness Frustration, and Novelty Frustration demonstrated solid stability. Competence Satisfaction and Novelty Satisfaction indicated moderate reliability. Overall, these findings confirm that responses to the instrument remain consistent over time.

The global fit indices for the measurement models are presented in Table 3. Initially, a confirmatory factor analysis was conducted. The results indicated an acceptable fit to the data (see Table 3). However, to better account for the conceptual proximity between basic psychological needs, an exploratory structural equation modeling approach using target rotation was evaluated. The exploratory structural equation model demonstrated a superior fit compared to the restricted confirmatory factor analysis framework. These results suggest that allowing for small, theoretically expected cross-loadings provides a more precise representation of the BPNSFNS structure in the exercise context.

Table 4 presents the standardized factor loadings and item reliability (λ^2^) for the BPNSFNS based on the confirmatory factor analysis model. All items demonstrated statistically significant factor loadings (*p* < 0.001). Most loadings exceeded the 0.50 threshold, indicating that the items are robust indicators of their respective latent constructs. Although Item 1 (“I feel a sense of choice and freedom in the things I undertake”) displayed a loading below 0.50 (FL = 0.39; λ^2^= 0.15), it was retained to preserve the theoretical integrity of the original scale. Its retention is justified by the strong global fit of the overall measurement model and its significant contribution to the autonomy satisfaction factor. Standardized loadings for the needs satisfaction ranged from 0.39 to 0.86, while loadings for the needs frustration ranged from 0.58 to 0.90, confirming the structural validity of the BPNSFNS.

Table 5 presents the composite reliability coefficients, average variance extracted values, and the discriminant validity matrix for the BPNSFNS. Internal consistency was confirmed across all dimensions, with composite reliability coefficients values ranging from 0.78 to 0.90, exceeding the recommended 0.70 threshold. Regarding convergent validity, average variance extracted values were above 0.50 for most subscales, indicating that the factors explain most of the variance in their respective items. Although autonomy satisfaction yielded an average variance extracted value of 0.48, it was deemed acceptable due to its strong composite reliability and the solid theoretical foundation of its indicators. Discriminant validity was assessed using a dual-criterion approach. Under the Fornell–Larcker criterion, despite some high latent correlations (e.g., between autonomy satisfaction and competence satisfaction, r = 0.79), the supplemental analysis of Heterotrait–Monotrait ratios provided robust evidence of construct distinctiveness. All Heterotrait–Monotrait ratios values remained below the conservative 0.85 threshold, with the highest ratio observed between autonomy satisfaction and novelty satisfaction (HTMT = 0.83). These findings, integrated with the superior fit of the exploratory structural equation modeling, seem to confirm that the eight dimensions of the BPNSFNS represent distinct motivational constructs within the exercise context.

To examine nomological validity, a structural equation modeling analysis was conducted using the ESEM-within-SEM framework. The eight basic psychological needs were defined as independent variables, while exercise enjoyment and satisfaction with life were included as outcome variables. The structural model demonstrated an excellent fit to the data (see Table 3). The model accounted for a substantial proportion of variance in the dependent variables, explaining 58% of enjoyment and 45% of satisfaction with life. Standardized path coefficients (β) and their respective 95% confidence intervals are summarized in Table 6. Regarding exercise enjoyment, significant positive associations were found with competence satisfaction and novelty satisfaction, whereas autonomy frustration and novelty frustration were significantly and negatively associated with this outcome. Autonomy, relatedness, and novelty satisfaction were positively and significantly associated with life satisfaction. Competence frustration was significantly and negatively associated with satisfaction with life. Overall, the patterns of association were consistent with theoretical expectations, supporting the nomological validity of the BPNSFNS. See Table 6 for details.

## 4. Discussion

The aim of this study was to translate and validate the Basic Psychological Need Satisfaction and Frustration Scale (BPNSFS) into Portuguese, specifically incorporating the need for novelty as proposed by González-Cutre et al. [18,20]. Given the framework of Self-Determination Theory, testing the psychometric robustness of novelty in the exercise domain is crucial for its recognition as a candidate basic psychological need. Our results indicate that the 35-item scale possesses strong psychometric properties, demonstrating good reliability and an improved fit through Exploratory Structural Equation Modeling (ESEM).

The validated instrument has been shown to be consistent over time, with an ICC ranging from 0.75 to 0.83, indicating acceptable to good reliability over time. These values are consistent with those reported by Filipe Rodrigues et al. [24], reinforcing the stability of the construct measurement in Portuguese exercisers.

Confirmatory factor analysis also revealed an acceptable fit, and the exploratory structural equation model showed a better fit than the confirmatory factor analysis, reinforcing the model’s accuracy and the possibility of bias in the estimation of correlations between factors and structural relationships. This finding is particularly significant when compared with the study by Filipe Rodrigues et al. [24], which employed a confirmatory factor analysis (CFA) model that yielded acceptable, albeit less than ideal, fit indices for the six-factor structure. The superior performance observed in the present study suggests that the inclusion of cross-loadings using ESEM may allow for a more accurate representation of the multidimensional nature of psychological needs, particularly when an additional construct such as novelty is incorporated.

A note regarding the analysis of factor loadings: these are generally adequate, but item 1 is below 0.50; nevertheless, it was retained to preserve the integrity and balance of the original scale. In line with suggestions made by Hair et al. [39], factor loadings below cutoff values should be considered for elimination. But this suggestion was only a guideline [39]. Other considerations are that this item enhances content validity, removing the item does not improve model fit, and the overall measurement model exhibits good fit. Therefore, we retained Item 1 so the scale would remain as close as possible to the original version like previously study [24].

Internal consistency presented no problems, nor did convergent validity in general.

It is in discriminant validity that the most concerning results appear to emerge, with some rather high latent correlations, which is once again unsurprising considering the theory and the way in which the different constructs complement and interconnect. However, when calculating Heterotrait–Monotrait ratios, all remain below the threshold (0.85), providing robust distinctiveness between constructs [52].

Central to the validation of a new need is its predictive efficacy for well-being. According to Self-Determination Theory, the satisfaction of basic needs leads to growth, while their frustration culminates in well-being [11]. Our findings support this, showing that novelty satisfaction, alongside autonomy, competence, and relatedness, are positively associated with enjoyment and satisfaction with life. In reverse, the frustration of these needs was associated with diminished well-being. These results reinforce the role of novelty as a psychological need that prevents monotony and raises sustained engagement in exercise [18,27,55]. However, it is important to note that these findings are based on a cross-sectional design and a convenience sample of fitness centre members, which limits the ability to establish causal relationships and the generalizability of the results to other populations.

### 4.1. Limitations

The present study is pioneering in the Portuguese context by examining novelty as a basic psychological need. Consequently, the absence of similar national studies, tied to the relative scarcity of international research on this construct, represents a limitation. However, these robust findings offer novel insights into the field, providing a validated instrument for national researchers to further explore this domain.

Despite its contributions, several limitations must be acknowledged. First, the cross-sectional design excludes definitive conclusions regarding causality, thus, the positive relationships observed should be interpreted as associations rather than causal links.

Second, the heterogeneity of the sample, including individuals exercising independently, with personal trainers, or in group classes, may influence the results. The interpersonal context of the exercise setting likely impacts the salience of specific needs, for instance, group environments might prioritize relatedness, whereas a personal trainer may differentially affect perceptions of autonomy and competence. As these contextual variations were not controlled for, they may have introduced unobserved variance. Future research should employ multi-group analyses to explore how different exercise modalities moderate the relationships between novelty and other psychological constructs.

Third, the use of a convenience sample from specific fitness centres may limit the generalizability of these findings to the broader population of exercisers.

Fourth, must be acknowledged that the Autonomy Satisfaction subscale has an Average Variance Extracted of 0.48, which is slightly below the conventional 0.50 threshold. While this is a subscale-level limitation, the construct was retained due to its strong composite reliability (0.78) and the essential theoretical role of its indicators within the Self-Determination Theory framework.

Furthermore, as the sample consisted exclusively of gym exercisers, the findings regarding the novelty construct may not be generalizable to clinical populations or sedentary individuals, who may exhibit different motivational dynamics.

Finally, given the emphasis of Self-Determination Theory on mediation, future research should expand on our findings by testing indirect effects (e.g., needs → enjoyment → life satisfaction) to further elucidate these complex pathways.

### 4.2. Practical Implications

The development and validation of this new scale in Portuguese is significant, as it provides another important tool in studies related to motivation and behavioral change.

The importance of the results of this study also lies in the fact that another step has been taken in the development of a new construct (novelty), in addition to the three already accepted and well-defined basic psychological needs (autonomy, competence and relatedness) that already are part of self-determination theory.

As this theory is of great importance and is used in different areas of knowledge, this study added value for a better understanding of human motivation and how to work with it. In the context of physical exercise and the Portuguese reality in particular, the added value is quite evident, since it is a fundamental area for human health, with proven benefits, both in prevention and as an adjuvant to therapy in various pathologies, and yet it is something with so little adherence in the Portuguese population.

## 5. Conclusions

The present study successfully translated and validated the Portuguese version of the Basic Psychological Need Satisfaction and Frustration Scale (BPNSFS) by integrating novelty as a distinct dimension. Through the application of Exploratory Structural Equation Modeling, factor structure demonstrated robust psychometric properties; internal consistency; and well-adjusted convergent, discriminant and nomological validity. These results confirm that the scale is a reliable and valid instrument for assessing psychological needs in the Portuguese exercise population.

Furthermore, our findings reinforce the conceptual proposal of novelty as a basic psychological need within the exercise context. By demonstrating that novelty satisfaction is intrinsically linked to increased enjoyment and satisfaction with life, while its frustration leads to diminished well-being, this study fulfils the essential criteria for identifying a psychological need. While this research is constrained by its cross-sectional nature, it aligns with emerging international literature suggesting that novelty operates similarly to the three traditional needs (autonomy, competence, and relatedness).

## Figures and Tables

**Table 1:** Sociodemographic and exercise characteristics of the sample (*N* = 500).

Variable	Frequency (n)	Percentage (%)	M	SD
Sex				
Female	263	52.6	-	-
Male	237	47.4	-	-
Activity Type				
Resistance Training	248	49.6	-	-
Group Classes	194	38.8	-	-
Personal Training	58	11.6	-	-
Age (years)	-	-	33.76	12.94
Weekly Frequency	-	-	3.44	1.44
Session Duration (min)	-	-	64.60	24.30

Note: *N* = total sample size; *n* = frequency; % = percentage; *M* = Mean; *SD* = Standard Deviation; min = minutes.

**Table 2:** Intraclass correlation coefficients for the BPNSFNS factors.

Factors	ICC	IC 95%
Autonomy Satisfaction	0.75	0.60, 0.85
Autonomy Frustration	0.76	0.61, 0.86
Competence Satisfaction	0.65	0.46, 0.79
Competence Frustration	0.78	0.64, 0.87
Relatedness Satisfaction	0.83	0.72, 0.90
Relatedness Frustration	0.80	0.67, 0,88
Novelty Satisfaction	0.72	0.55, 0,83
Novelty Frustration	0.81	0.69, 0.89

Note: All values are significant at *p* < 0.01.

**Table 3:** Global fit indices for the measurement and structural models.

Model	χ^2^	df	CFI	TLI	RMSEA [90% CI]	WRMR
CFA	1075.05	532	0.96	0.96	0.05 [0.04, 0.05]	1.00
ESEM	558.06	343	0.98	0.97	0.04 [0.03, 0.04]	0.48
SEM	1569.91	846	0.98	0.97	0.04 [0.04, 0.05]	0.78

Note: χ^2^ = chi-square; df = degrees of freedom; CFI = Comparative Fit Index; TLI = Tucker–Lewis Index; RMSEA = Root Mean Square Error of Approximation; CI = Confidence Interval; WRMR = Weighted Root Mean Square Residual; SEM = Structural Equation Modelling for nomological validity.

**Table 4:** Standardized factor loadings and item reliability for the factor models.

Factor/Item	CFA (λ)	CFA (λ^2^)	ESEM (λ)	ESEM (λ^2^)
Autonomy Satisfaction				
Item 1	0.389	0.152	0.270	0.358
Item 9	0.728	0.530	0.547	0.663
Item 17	0.778	0.605	0.532	0.595
Item 25	0.796	0.633	0.500	0.593
Autonomy Frustration				
Item 2	0.581	0.337	0.538	0.449
Item 10	0.732	0.535	0.611	0.593
Item 18	0.722	0.522	0.435	0.515
Item 26	0.903	0.815	0.527	0.769
Competence Satisfaction				
Item 5	0.801	0.642	0.594	0.686
Item 13	0.827	0.683	0.665	0.713
Item 21	0.836	0.699	0.462	0.658
Item 29	0.673	0.453	0.578	0.559
Competence Frustration				
Item 6	0.764	0.583	0.486	0.579
Item 14	0.708	0.501	0.522	0.581
Item 22	0.792	0.627	0.477	0.666
Item 30	0.712	0.506	0.447	0.515
Relatedness Satisfaction				
Item 3	0.665	0.442	0.623	0.511
Item 11	0.855	0.731	0.797	0.784
Item 19	0.783	0.613	0.655	0.609
Item 27	0.693	0.480	0.468	0.572
Relatedness Frustration				
Item 4	0.769	0.592	0.211	0.622
Item 12	0.814	0.663	0.486	0.681
Item 20	0.805	0.648	0.617	0.722
Item 28	0.728	0.531	0.448	0.521
Novelty Satisfaction				
Item 7	0.786	0.617	0.505	0.612
Item 15	0.772	0.596	0.865	0.699
Item 23	0.798	0.636	0.769	0.693
Item 31	0.721	0.520	0.628	0.607
Item 33	0.823	0.677	0.600	0.629
Item 35	0.562	0.316	0.640	0.382
Novelty Frustration				
Item 8	0.790	0.624	0.754	0.704
Item 16	0.774	0.598	0.587	0.592
Item 24	0.800	0.641	0.586	0.627
Item 32	0.740	0.547	0.794	0.657
Item 34	0.899	0.807	0.801	0.809

Note: λ = Standardized factor loading; λ^2^ = Item reliability (R-square). CFA = Confirmatory Factor Analysis; ESEM = Exploratory Structural Equation Modeling. All target loadings are significant at *p* < 0.001.

**Table 5:** Composite reliability, convergent validity, and discriminant validity matrix.

Factor	CR	AVE	1	2	3	4	5	6	7	8
1 Autonomy Satisfaction	0.78	0.48	(0.69)	0.65	0.80	0.52	0.76	0.48	0.83	0.45
2 Autonomy Frustration	0.83	0.55	−0.59	(0.74)	0.54	0.82	0.42	0.82	0.50	0.83
3 Competence Satisfaction	0.87	0.62	0.79	−0.48	(0.79)	0.79	0.62	0.49	0.68	0.34
4 Competence Frustration	0.83	0.56	−0.46	0.72	−0.69	(0.75)	0.35	0.82	0.33	0.67
5 Relatedness Satisfaction	0.84	0.57	0.68	−0.38	0.56	−0.33	(0.75)	0.71	0.59	0.38
6 Relatedness Frustration	0.86	0.61	−0.44	0.76	−0.45	0.77	−0.66	(0.78)	0.34	0.65
7 Novelty Satisfaction	0.88	0.56	0.75	−0.46	0.62	−0.31	0.54	−0.32	(0.75)	0.65
8 Novelty Frustration	0.90	0.64	−0.42	0.77	−0.32	0.64	−0.36	0.62	−0.60	(0.80)

Note: CR = Composite Reliability; AVE = Average Variance Extracted. Values on the diagonal (in parentheses) represent the square root of the AVE. Values below the diagonal represent standardized latent correlations. Values above the diagonal represent the Heterotrait–Monotrait ratios.

**Table 6:** Standardized path coefficients and significance for the nomological validity model.

Path	β	95% CI	*p*-Value
Dependent Variable (Enjoyment) (R^2^ = 0.58)			
Autonomy Satisfaction → Enjoyment	0.01	[−0.11, 0.13]	0.88
Competence Satisfaction → Enjoyment	0.44	[0.32, 0.56]	<0.01
Relatedness Satisfaction → Enjoyment	0.13	[0.00, 0.25]	0.06
Novelty Satisfaction → Enjoyment	0.15	[0.01, 0.28]	0.03
Autonomy Frustration → Enjoyment	−0.39	[−0.51, −0.28]	<0.01
Competence Frustration → Enjoyment	0.30	[0.16, 0.44]	<0.01
Relatedness Frustration → Enjoyment	−0.00	[−0.19, 0.18]	0.98
Novelty Frustration → Enjoyment	−0.14	[−0.26, −0.01]	0.03
Dependent Variable (Satisfaction with life) (R^2^ = 0.45)			
Autonomy Satisfaction → Satisfaction with life	0.18	[0.01, 0.35]	0.04
Competence Satisfaction → Satisfaction with life	0.05	[−0.09, 0.19]	0.48
Relatedness Satisfaction → Satisfaction with life	0.26	[0.14, 0.39]	<0.01
Novelty Satisfaction → Satisfaction with life	0.20	[0.07, 0.33]	<0.01
Autonomy Frustration → Satisfaction with life	0.05	[−0.09, 0.20]	0.46
Competence Frustration → Satisfaction with life	−0.36	[−0.47, −0.25]	<0.01
Relatedness Frustration → Satisfaction with life	0.15	[0.01, 0.28]	0.04
Novelty Frustration → Satisfaction with life	−0.07	[−0.19, 0.05]	0.26

Note: β = standardized path coefficient; CI = confidence interval. R^2^ represents the proportion of explained variance for each outcome.

## Data Availability

The data that support the findings of this study are available from the corresponding author upon reasonable request.

## References

[1] Kunutsor S.K., Laukkanen J.A. (2024). Physical Activity, Exercise and Adverse Cardiovascular Outcomes in Individuals with Pre-Existing Cardiovascular Disease: A Narrative Review. Expert Rev. Cardiovasc. Ther..

[2] Loro F.L., Ostolin T.L.V.D.P. (2023). Atividade física, comportamento sedentário e saúde da mulher: Um mapa de evidências. Rev. Bras. Atividade Física Saúde.

[3] European Union Sport and Physical Activity–European Sources Online 2022. https://www.europeansources.info/record/sport-and-physical-activity/.

[4] Orr J. (1991). Piaget’s Theory of Cognitive Development May Be Useful in Deciding What to Teach and How to Teach It. Nurse Educ. Today.

[5] Wolff P.H. (1963). Developmental and motivational concepts in piaget’s sensorimotor theory of intelligence. J. Am. Acad. Child Psychiatry.

[6] Deci E.L., Ryan R.M. (2000). The “What” and “Why” of Goal Pursuits: Human Needs and the Self-Determination of Behavior. Psychol. Inq..

[7] Deci E., Ryan R.M. (1985). Intrinsic Motivation and Self-Determination in Human Behavior.

[8] Teixeira P.J., Carraça E.V., Markland D., Silva M.N., Ryan R.M. (2012). Exercise, Physical Activity, and Self-Determination Theory: A Systematic Review. Int. J. Behav. Nutr. Phys. Act..

[9] Vansteenkiste M., Ryan R.M. (2013). On Psychological Growth and Vulnerability: Basic Psychological Need Satisfaction and Need Frustration as a Unifying Principle. J. Psychother. Integr..

[10] Silva M.N., Markland D., Carraça E.V., Vieira P.N., Coutinho S.R., Minderico C.S., Matos M.G., Sardinha L.B., Teixeira P.J. (2011). Exercise Autonomous Motivation Predicts 3-Yr Weight Loss in Women. Med. Sci. Sports Exerc..

[11] Ryan R.M., Deci E.L. (2017). Self-Determination Theory: Basic Psychological Needs in Motivation, Development, and Wellness.

[12] Bartholomew K.J., Ntoumanis N., Ryan R.M., Bosch J.A., Thøgersen-Ntoumani C. (2011). Self-Determination Theory and Diminished Functioning: The Role of Interpersonal Control and Psychological Need Thwarting. Pers. Soc. Psychol. Bull..

[13] Arnett J. (1994). Sensation Seeking: A New Conceptualization and a New Scale. Personal. Individ. Differ..

[14] Kashdan T., Disabato D., Goodman F., Mcknight P. (2019). The Five-Dimensional Curiosity Scale Revised (5DCR): Briefer Subscales While Separating General Overt and Covert Social Curiosity. Personal. Individ. Differ..

[15] Sylvester B.D., Standage M., Dowd A.J., Martin L.J., Sweet S.N., Beauchamp M.R. (2014). Perceived Variety, Psychological Needs Satisfaction and Exercise-Related Well-Being. Psychol. Health.

[16] Bagheri L., Milyavskaya M. (2020). Novelty–Variety as a Candidate Basic Psychological Need: New Evidence across Three Studies. Motiv. Emot..

[17] Vansteenkiste M., Ryan R.M., Soenens B. (2020). Basic Psychological Need Theory: Advancements, Critical Themes, and Future Directions. Motiv. Emot..

[18] González-Cutre D., Sicilia Á., Sierra A.C., Ferriz R., Hagger M.S. (2016). Understanding the Need for Novelty from the Perspective of Self-Determination Theory. Personal. Individ. Differ..

[19] Trigueros R., Mínguez L.A., González-Bernal J.J., Aguilar-Parra J.M., Padilla D., Álvarez J.F. (2019). Validation of the Satisfaction Scale of Basic Psychological Needs in Physical Education with the Incorporation of the Novelty in the Spanish Context. Sustainability.

[20] González-Cutre D., Romero-Elías M., Jiménez-Loaisa A., Beltrán-Carrillo V.J., Hagger M.S. (2020). Testing the Need for Novelty as a Candidate Need in Basic Psychological Needs Theory. Motiv. Emot..

[21] Trigueros R., Álvarez J.F., Cangas A.J., Aguilar-Parra J.M., Méndez-Aguado C., Rocamora P., López-Liria R. (2020). Validation of the Scale of Basic Psychological Needs towards Physical Exercise, with the Inclusion of Novelty. Int. J. Environ. Res. Public Health.

[22] Ryan R.M., Deci E.L. (2002). Overview of Self-Determination Theory: An Organismic-Dialectical Perspective. Handbook of Self-Determination Research.

[23] Fierro-Suero S., Almagro B.J., Sáenz-López P., Carmona-Márquez J. (2020). Perceived Novelty Support and Psychological Needs Satisfaction in Physical Education. Int. J. Environ. Res. Public Health.

[24] Rodrigues F., Hair J.F., Neiva H.P., Teixeira D.S., Cid L., Monteiro D. (2019). The Basic Psychological Need Satisfaction and Frustration Scale in Exercise (BPNSFS-E): Validity, Reliability, and Gender Invariance in Portuguese Exercisers. Percept. Mot. Skills.

[25] Aibar A., Abós Á., García-González L., González-Cutre D., Sevil-Serrano J. (2021). Understanding Students’ Novelty Satisfaction in Physical Education: Associations with Need-Supportive Teaching Style and Physical Activity Intention. Eur. Phys. Educ. Rev..

[26] Benlahcene A., Kaur A., Awang-Hashim R. (2020). Basic Psychological Needs Satisfaction and Student Engagement: The Importance of Novelty Satisfaction. J. Appl. Res. High. Educ..

[27] Birdsell B. (2018). Understanding Students’ Psychological Needs in an English Learning Context. J. Lib. Arts Dev. Pract..

[28] Fernández-Espínola C., Almagro B.J., Tamayo-Fajardo J.A., Sáenz-López P. (2020). Complementing the Self-Determination Theory With the Need for Novelty: Motivation and Intention to Be Physically Active in Physical Education Students. Front. Psychol..

[29] Koka A., Tilga H., Hein V., Kalajas-Tilga H., Raudsepp L. (2021). A Multidimensional Approach to Perceived Teachers’ Autonomy Support and Its Relationship with Intrinsic Motivation of Students in Physical Education. Int. J. Sport Psychol..

[30] Stoa R., Chu T.L.A. (2023). An Argument for Implementing and Testing Novelty in the Classroom. Scholarsh. Teach. Learn. Psychol..

[31] Berki T., Csányi T., Tóth L. (2024). Associations of Physical Activity and Physical Education Enjoyment with Self-Concept Domains among Hungarian Adolescents. BMC Psychol..

[32] Westerskov Dalgas B., Elmose-Østerlund K., Bredahl T.V.G. (2024). Exploring Basic Psychological Needs within and across Domains of Physical Activity. Int. J. Qual. Stud. Health Well-Being.

[33] Chen C., Weyland S., Fritsch J., Woll A., Niessner C., Burchartz A., Schmidt S.C.E., Jekauc D. (2021). A Short Version of the Physical Activity Enjoyment Scale: Development and Psychometric Properties. Int. J. Environ. Res. Public Health.

[34] Raedeke T.D. (2007). The Relationship Between Enjoyment and Affective Responses to Exercise. J. Appl. Sport Psychol..

[35] Ntoumanis N., Moller A.C. (2025). Self-Determination Theory Informed Research for Promoting Physical Activity: Contributions, Debates, and Future Directions. Psychol. Sport Exerc..

[36] Navarro-Patón R., Rodríguez-Negro J., Muíño-Piñeiro M., Mecías-Calvo M. (2024). Gender and Educational Stage Differences in Motivation, Basic Psychological Needs and Enjoyment: Evidence from Physical Education Classes. Children.

[37] Huta V., Waterman A.S. (2014). Eudaimonia and Its Distinction from Hedonia: Developing a Classification and Terminology for Understanding Conceptual and Operational Definitions. J. Happiness Stud..

[38] Soper D. A-Priori Sample Size Calculator for Structural Equation Models [Software] 2023. https://www.danielsoper.com/statcalc.

[39] Hair J., Babin B., Anderson R., Black W. (2019). Multivariate Data Analysis.

[40] Kline R.B. (2016). Principles and Practice of Structural Equation Modeling.

[41] Brislin R. (1980). Translation and Content Analysis of Oral and Written Material. Handbook of Cross-Cultural Psychology: Methodology.

[42] Banville D., Desrosiers P., Genet-Volet Y. (2000). Translating Questionnaires and Inventories Using a Cross-Cultural Translation Technique. J. Teach. Phys. Educ..

[43] Cid L., Monteiro D., Teixeira D.S., Evmenenko A., Andrade A., Bento T., Vitorino A., Couto N., Rodrigues F. (2022). Assessment in Sport and Exercise Psychology: Considerations and Recommendations for Translation and Validation of Questionnaires. Front. Psychol..

[44] Teques P., Calmeiro L., Silva C., Borrego C. (2020). Validation and Adaptation of the Physical Activity Enjoyment Scale (PACES) in Fitness Group Exercisers. J. Sport Health Sci..

[45] Neto F. (1993). The Satisfaction with Life Scale: Psychometrics Properties in an Adolescent Sample. J. Youth Adolesc..

[46] Koo T.K., Li M.Y. (2016). A Guideline of Selecting and Reporting Intraclass Correlation Coefficients for Reliability Research. J. Chiropr. Med..

[47] Muthén L., Muthén B. (2017). Mplus: Statistical Analysis with Latent Variables: User’s Guide.

[48] Byrne B.M. (2016). Structural Equation Modeling with AMOS: Basic Concepts, Applications, and Programming.

[49] Marsh H.W., Hau K.-T., Wen Z. (2004). In Search of Golden Rules: Comment on Hypothesis-Testing Approaches to Setting Cutoff Values for Fit Indexes and Dangers in Overgeneralizing Hu and Bentler’s (1999) Findings. Struct. Equ. Model. A Multidiscip. J..

[50] Raykov T. (1997). Estimation of Composite Reliability for Congeneric Measures. Appl. Psychol. Meas..

[51] Fornell C., Larcker D.F. (1981). Evaluating Structural Equation Models with Unobservable Variables and Measurement Error. J. Mark. Res..

[52] Henseler J., Ringle C.M., Sarstedt M. (2015). A New Criterion for Assessing Discriminant Validity in Variance-Based Structural Equation Modeling. J. Acad. Mark. Sci..

[53] Asparouhov T., Muthén B. (2009). Exploratory Structural Equation Modeling. Struct. Equ. Model. A Multidiscip. J..

[54] Williams J., MacKinnon D.P. (2008). Resampling and Distribution of the Product Methods for Testing Indirect Effects in Complex Models. Struct. Equ. Model..

[55] Chatzisarantis N., Hagger M. (2007). Reflecting on the Past and Sketching the Future. Intrinsic Motivation and Self-Determination in Exercise and Sport.

